# Alveolar Bone Loss in Diabetic Patients: A Case–Control Study

**DOI:** 10.1055/s-0042-1758071

**Published:** 2022-12-15

**Authors:** Afsheen Tabassum

**Affiliations:** 1 Department of Preventive Dental Sciences, College of Dentistry, Imam Abdulrahman Bin Faisal University, Dammam, Kingdom of Saudi Arabia

**Keywords:** periodontitis, alveolar bone loss, diabetes mellitus, case–control study

## Abstract

**Objectives** Based on literature, very few case–control studies have been executed to confirm the relationship between diabetes mellitus and the severity of mean alveolar bone loss. Therefore, the aim was to assess the differences in mean alveolar bone loss among diabetic (type 2) and nondiabetic patients in the Saudi population.

**Materials and Methods** Nine-hundred eighty-two patient records were seen in this retrospective study. Patient demographic data and medical records were examined. The mean alveolar bone loss was measured in posterior teeth by calculating the distance from the base of cementoenamel junction to the alveolar crest using the bitewing radiographs. SPSS 20.0 was used for data analysis. An unpaired *t* -test was utilized to analyze the mean alveolar bone loss across multiple variables. *p* -Value less than or equal to 0.05 was contemplated as significant.

**Results** The overall mean alveolar bone loss for all 124 patients was 2.83 ± 1.13 mm. Diabetic patients had greater mean alveolar bone loss measured in millimeters than nondiabetic patients (3.07 ± 1.14mm vs. 2.59 ± 1.08mm, respectively), and the difference was significant ( *p* = 0.018). In terms of the severity of mean alveolar bone loss, diabetic patients experience statistically higher mean alveolar bone loss as compared with nondiabetic patients.

**Conclusion** In our study population, the overall mean alveolar bone loss prevalence was greater in diabetes patients than in nondiabetic individuals. According to the severity of bone loss, the distribution of moderate and severe periodontitis was higher in diabetic patients. To enhance patients' quality of life, the awareness and education among patients regarding the association among diabetes mellitus and oral health, particularly periodontal disease, should be promoted.

## Introduction

Diabetes mellitus (DM) is considered a major public concern. 1 2 Diabetes is categorized into two main types: type 1 (Insulin-dependent) and type 2 (Noninsulin-dependent). 3 Type 2 DM represents approximately 95% of diabetic cases. 4 Based on the International Diabetes Federation data, DM prevalence has sharply increased worldwide during the last few years and will triple in the following decade. 1 It is estimated that between 1992 and 2022, type 2 DM prevalence among Saudi adults aged 25 and older will increase significantly, from 8.5 to 39.5%. 5 6 Comorbid symptoms like nephropathy, neuropathy, and cardiovascular complications are ultimately brought on by DM. 7 Additionally, DM has been linked to several oral health-related disorders, including periodontal disease (PD), xerostomia, halitosis, fungal infections, and oral mucosal lesions. 3 8 9

PD is an oral infection that is distinguished by the loss of alveolar bone support around teeth. 10 11 Severe periodontitis can lead to tooth loss and affects approximately 5 to 20% of the world's adult population. The association between DM and periodontitis has been reported in the literature. 12 13 14 DM is linked to alveolar bone loss and is known as a risk factor for PD. 15 A higher clinical attachment loss (CAL), alveolar bone loss, a greater percentage of furcation lesions and tooth mobility are correlated with diabetes. 14 15 16

Persistent hyperglycemia causes extravagant immune responses in the presence of periodontal pathogens and is expected to be accountable for the increased risk and severity of alveolar hone loss in diabetic patients. 17 18 Diabetes has a significant impact on osteoclastogenesis and increases osteoblast apoptosis. 19 Nevertheless, a bidirectional relationship has been reported between periodontitis and DM. 20 PD negatively affects the glycemic control of the patients and it has been reported that host-derived inflammatory mediators released due to increasing bacterial load in PD play a vital role. 15

Furthermore, Heji et al reported that periodontal examination findings can help to identify patients at risk of undiagnosed diabetes or prediabetes. 21 By performing a comprehensive radiographic and periodontal examination, dental practitioners can identify patients who have a risk factor for diabetes, particularly if they have a family history of diabetes, and earlier referral to the medical can be established. This relationship must be understood not only by dentists but also by physicians who work to improve the lifestyle and well-being of diabetic patients. Matrooshi et al demonstrated that, despite having good knowledge, physicians seldom refer diabetic patients for appropriate periodontal care. 22

Based on the literature, very few case–control studies have been executed to confirm the relationship between DM and the severity of mean alveolar bone loss (MABL). Furthermore, no such study has been conducted in Saudi Arabia's Eastern province. There is a substantial need to investigate the differences in MABL among diabetic and nondiabetic individuals. This study aimed to evaluate the mean differences in alveolar bone loss among diabetic (type 2) and nondiabetic patients in the Saudi population.

## Materials and Methods

### Ethical Approval

Ethical approval for this retrospective study was acquired from the Institutional review board of Imam Abdulrahman bin Faisal University (IAU), Dammam, Saudi Arabia (IRB approval number: IRB:2020-02-109). This study was conducted according to the Helsinki Declaration guidelines.

### Patient Selection

The College of Dentistry, IAU, database was searched for patients who had a clinic visit in the last 3 years. Inclusion criteria for patient records were availability of complete intra, extraoral, and radiographic examinations of the patient.

#### Inclusion Criteria

Patient inclusion criteria for this study were (1) patient age (18 years old or older), (2) complete medical and dental records of the patients, and (3) Bitewing (BW) radiographs of the patients.

#### Exclusion Criteria

Following were the study's patient exclusion criteria: (1) patients with an incomplete medical history, (2) the patient's whose BW radiographs were missing, and (3) patients who did not have at least two adjacent teeth or whose interproximal space between teeth was narrow, and the crest of the bone was not visible. The information gathered includes the patient's age, gender, medical history, nationality, and radiographs.

### Radiographic Examination

In this study, BW radiographs were utilized to assess MABL. The BW radiographs were used to reduce angular distortion. Alveolar bone loss was calculated on the mesial and distal surfaces of the mandibular and maxillary posterior teeth using ImageJ software (Wayne Rasband, version 1.47, National Institutes of Health, Bethesda, Maryland, United States). Mean of mesial and distal bone loss was calculated and used for further analysis as MABL. All of the measurements were standardized using a radiograph of an implant of known diameter as a reference.

**Fig. 1:**
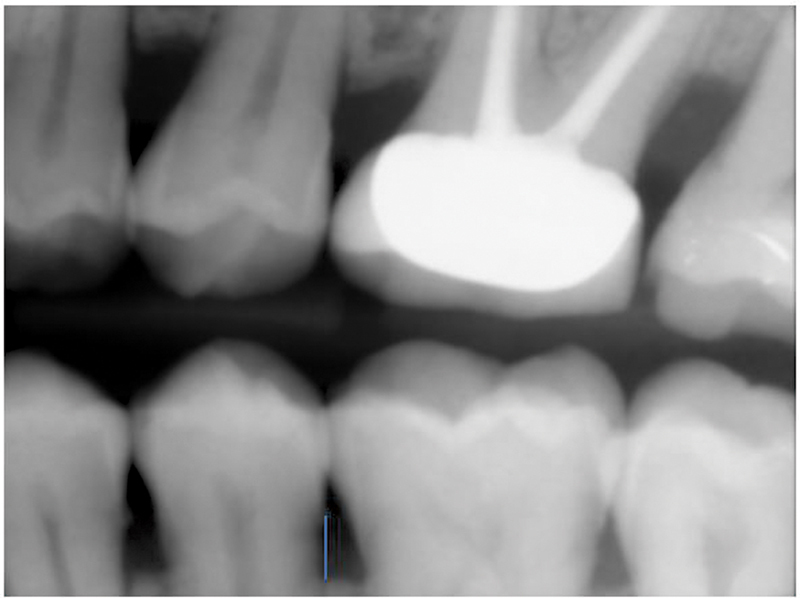
A bitewing radiographs of the patient for alveolar bone loss measurements. A straight line was sketched on the mesial and distal side of the tooth parallel to the long axis of each tooth from the most apical part of cementoenamel junction to the alveolar crest to measure the mean alveolar bone loss. A blue line is drawn at the distal aspect of the second premolar.

To calculate alveolar bone loss, a line was drafted along the long axis of tooth on the mesial and distal sides from apical part of the cementoenamel junction (CEJ) to the top of the alveolar crest ( Fig. 1 ). A distance greater than or equal to 2 mm between the CEJ and the alveolar crest was contemplated as a radiographic sign of interproximal bone loss. 13 14 15 17 The severity was categorized based on case definition by task force classification from the American Academy of Periodontology into normal (MABL< 2mm), mild (MABL >2mm ≤3mm), moderate (MABL >3mm ≤5mm), and severe periodontitis (MABL >5mm). 13

All the BW radiographs were examined in a dark room for accurate measurements. The convenient sample size was considered for this study.

### Statistical Analysis

SPSS 20.0 was used for data analysis (IBM product, Chicago, Illinois, United States). Gender, nationality, and age were among the categorical variables that were provided as frequencies and percentages. Age and bone loss measurements were included in the numerical data, which were displayed as mean standard deviation. Unpaired *t* -test was used to compare the MABL in different groups of teeth and maxilla versus mandible between diabetic patients and nondiabetic individuals. To evaluate effect of categorical variables, the MABL was compared in relation to gender, nationality, and age between the groups by employing unpaired *t* -test. A *p* -value less than or equal to 0.05 was considered significant.

## Results

**Fig. 2:**
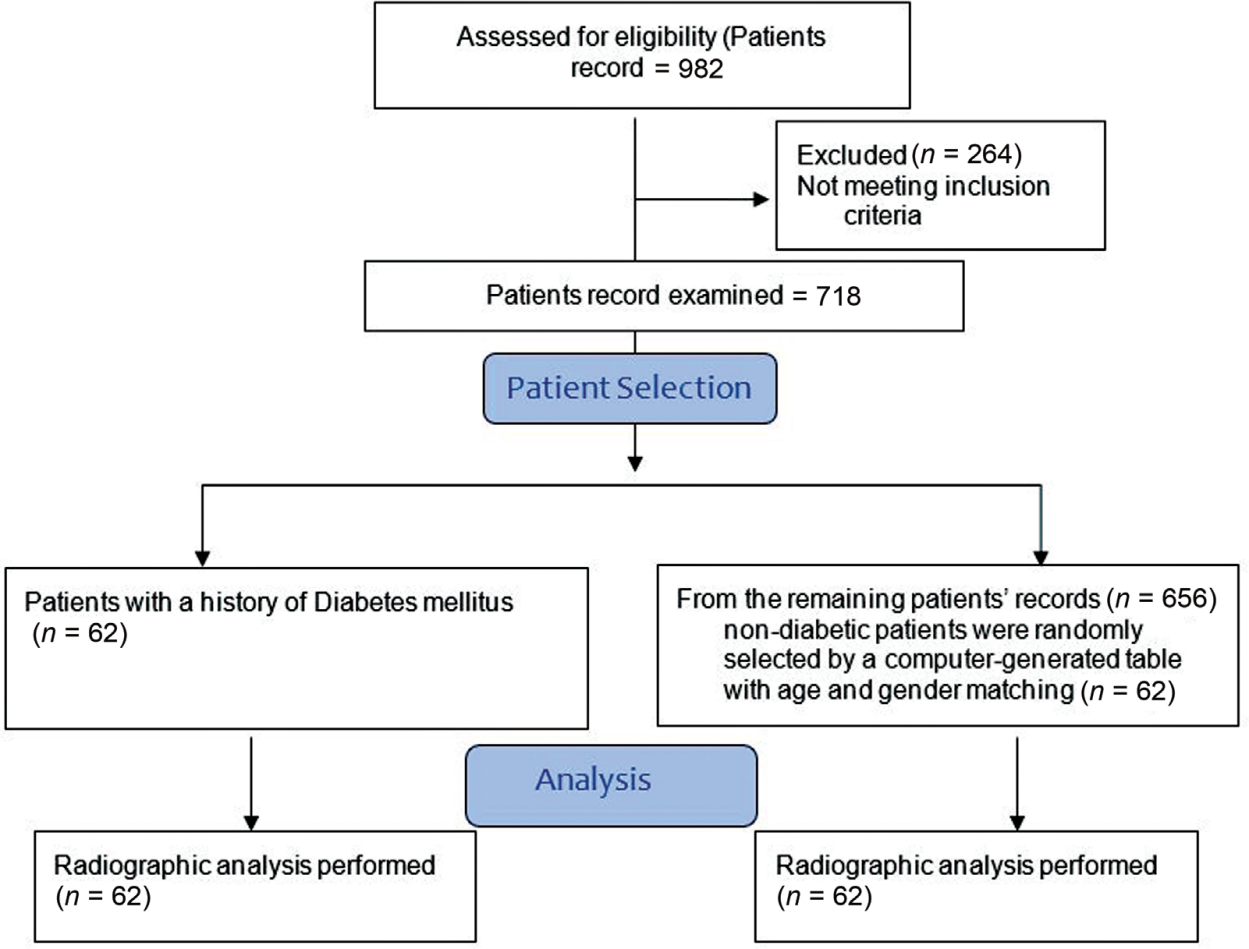
Flow diagram for patient selection.

The 982 patient records were examined and out of which 264 were excluded due to incomplete records. Seven-hundred eighteen records were examined and only 62 of these individuals, as indicated by their medical histories, had type 2 DM ( Fig. 2 ). Patients in the control group ( *n* = 62) were chosen randomly from age and gender-matched computer-generated table with no prior history of DM. The secondary matching criterion was nationality. The average age of all the patients was 48.02 ± 12.90. In the diabetic group, there were 40 Saudi (67%) and 22 non-Saudi nationals (33%). The patients' average age was 48.21 ± 13.03 years. There were 44 Saudi (71%) and 18 non-Saudi nationals (29%) in the control group. The patients' average age was 47.8 12.9 years. In both diabetic and control groups, there was 42 male (67.7%) versus 20 female patients (32.3%).

The overall MABL for all 124 patients was 2.83 ± 1.13 mm. Diabetic patients had greater MABL loss measured in millimeters than nondiabetic patients (3.07 ± 1.14 mm vs. 2.59 ± 1.08mm, respectively), and the difference was significant ( *p* = 0.018). In terms of the severity of MABL, diabetic patients experience statistically higher bone loss compared with nondiabetic patients. Table 1 represents the variations in MABL based on severity among diabetic and nondiabetic patients. The average prevalence of MABL was significantly greater in diabetic (85.5%) than nondiabetic patients (71%).

**Table 1:** Severity and prevalence of mean alveolar bone loss in diabetic and nondiabetic patients

The severity of alveolar bone loss	Nondiabetic ( *n* = 62)	Diabetic ( *n* = 62)	*p* -Value
	*n* (%)	*n* (%)	
Normal ( *n* = 27)	18 (29)	9 (14.5)	0.043 ^a^
Mild ( *n* = 59)	31 (50)	28 (45.2)	
Moderate ( *n* = 31)	10 (16.1)	21 (33.8)	
Severe ( *n* = 7)	3 (4.8)	4 (6.5)	
Alveolar bone loss prevalence			0.028 ^a^
Normal	18 (29)	9 (14.5)	
Alveolar bone loss	44 (71)	53 (85.5)	

*The severity of bone loss: normal (<2 mm), mild (2–3 mm), moderate (>3–5 mm), severe (>5 mm).*^a^ Statistically significant results at a 5% level of significance.

**Fig. 3:**
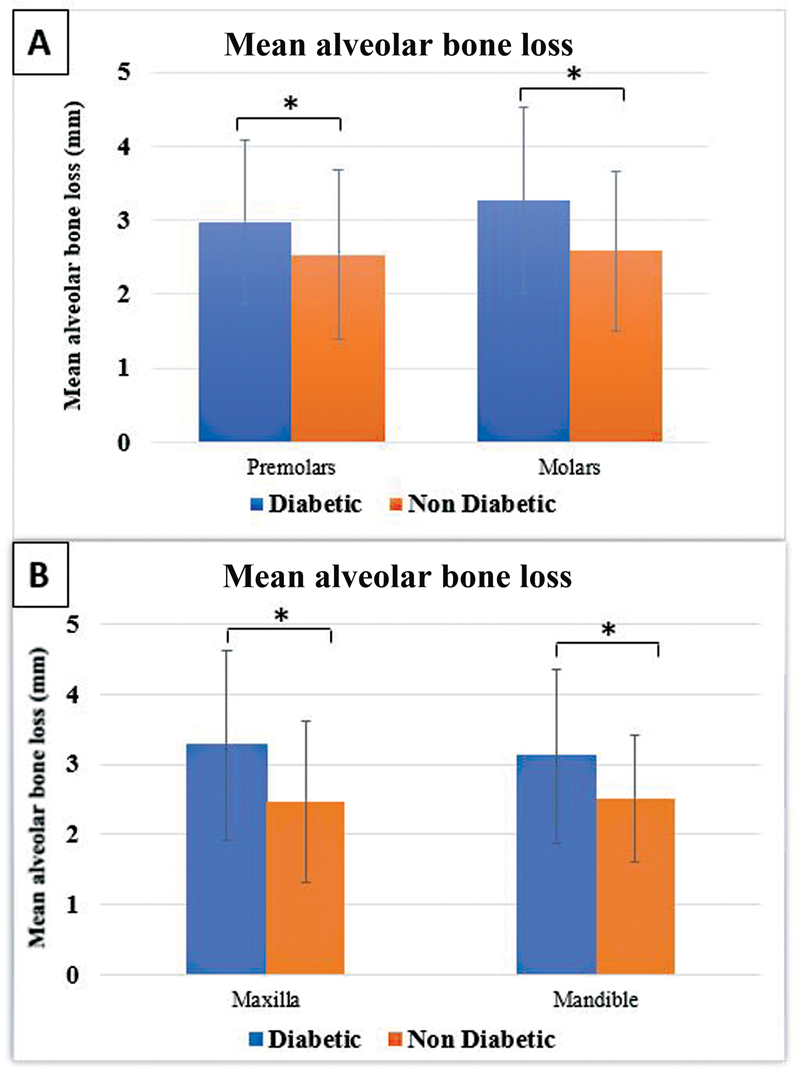
( **A** ) Differences in mean alveolar bone loss in premolars and molars among diabetic and nondiabetic patients. ( **B** ) Differences in mean alveolar bone loss in maxillary and mandibular teeth among diabetic and nondiabetic patients. *Statistically significant results at a 5% level of significance.

The MABL in various groups of teeth in diabetic patients versus nondiabetic patients is shown in Fig. 3A and Table 2 . There was a significantly greater MABL in both premolar ( *p* = 0.039) and molar ( *p* = 0.003) groups of teeth in diabetic versus nondiabetic subjects. When the teeth were divided into groups of maxillary and mandibular teeth, a statistically significant difference was observed showing that diabetic patients had a higher MABL in both the maxilla ( *p* = 0.002) and the mandible ( *p* = 0.006) ( Fig. 3B ).

**Table 2:** Mean alveolar bone loss in different groups of teeth in nondiabetic and diabetic patients

Groups of teeth	Diabetic ( *n* = 62)	Nondiabetic ( *n* = 62)	*p* -Value
Maxillary molars	3.21 ± 1.34	2.42 ± 1.75	0.003 a
Mandibular molars	2.9 ± 1.28	2.36 ± 0.91	0.022 a
Maxillary premolars	3.4 ± 1.51	2.54 ± 1.29	0.006 a
Mandibular premolars	3.27 ± 1.33	2.5 ± 0.89	0.002 a

a Statistically significant results at a 5% level of significance.

Gender-wise differences in overall MABL among the diabetic and nondiabetic individuals were found to be significant for male patients, but nonsignificant for female patients ( Table 3 ). Age-wise differences in MABL among the diabetic and nondiabetic individuals were found to be higher for diabetic patients in both age groups; however, statistically it was determined to be nonsignificant ( Table 3 ). Furthermore, nationality-wise differences of mean bone loss among the diabetic and nondiabetic patients were higher in the Saudi diabetic population; however, the differences were observed to be nonsignificant ( Table 3 ).

**Table 3:** Gender, age, and nationality wise differences of mean alveolar bone loss among diabetic and nondiabetic patients

Groups of teeth	Diabetic Mean ± SD ( *n* )	Nondiabetic Mean ± SD ( *n* )	*p* -Value
**Gender**			
Male	3.11 ± 1.18 (41)	2.42 ± 0.86 (40)	0.003 a
Female	2.96 ± 1.02 (21)	2.9 ± 1.36 (22)	0.88
**Age**			
Below 50	3.0963 ± 1.20 (30)	2.5830 ± 1.15 (30)	0.10
Above 50	3.0573 ± 1.10 (32)	2.6041 ± 1.03 (32)	0.09
**Nationality**			
Saudi	3.20 ± 1.15 (40)	2.7070 ± 1.19 (44)	0.061
Non-Saudi	2.85 ± 1.09 (22)	2.3172 ± 0.69 (18)	0.078

Abbreviation: SD, standard deviation.

a Statistically significant results at a 5% level of significance.

## Discussion

This study found a link between type 2 diabetes and MABL in a cohesive study population. This relationship remained consistent regardless of the severity of mean alveolar bone loss, its prevalence, site (maxilla or mandible), or position of the teeth. Gender, age, and ethnicity statistical analyses were performed in this study to account for the effect of age, gender, and ethnicity. This allowed for a more accurate assessment of the relationship between MABL and type 2 diabetes compared with nondiabetic subjects.

In this study, a case–control study design was used due to its distinct advantages over other study designs. Case–control studies are relatively reliable and low cost. The controls were chosen with age and gender as primary matching criteria and nationality as a secondary matching criterion' between the test and control groups. Marginal bone loss was assessed using BW radiographs. Other diagnostic tools, such as periapical and panoramic radiographs, have been used to detect bone loss, but they provide limited diagnostic information. Several studies have confirmed that BW radiography is still the most effective diagnostic radiography for crestal bone examination. 23 24 It is a faster, less expensive method with a lower radiation dose.

Our findings are consistent with previous research. Diabetes is a known risk factor for PD. PD is common (34–68%) in diabetic patients, as well as increased pocket probing depths and CAL. 25 26 Patients whose glycemic control is poor have a higher risk of PD compared with healthy individuals. 27 Periodontitis is considered a sixth oral manifestation of diabetes. 15 In addition, diabetic patients have a greater susceptibility to periodontitis development and advancement in comparison to healthy individuals. 28 29

Antibodies produced against microorganisms in periodontal tissues and predisposing genetic factors lay the foundation for an autoimmune role in PD pathogenesis. 30 Diabetes causes autoimmunity because of a breakdown in the normal defense process caused by by-products of tissue breakdown and chronic infection. PD and diabetes are linked epidemiologically because both conditions have autoimmune components. 31 Because PD and diabetes have a bidirectional relationship, proper periodontal care can improve metabolic control in DM patients. 32 The results of a meta-analysis that included nine randomized clinical trials confirmed the effectiveness of nonsurgical periodontal therapy in glycemic control and a moderate decrease in hemoglobin A1c among diabetic patients was observed. 33

Concerning the severity and prevalence of MABL, this study showed that 50 and 45.2% of participants had mild, 16.1 and 33.8% had moderate, and only 4.8 and 6.5% had severe mean alveolar bone loss among nondiabetic and diabetic individuals, respectively. Correspondingly, Zahid et al observed a prevalence of mild, moderate, and severe alveolar bone loss comparable to our results, that is, 57.4% of the participants with mild, 36.6% moderate, and 4.95% severe alveolar bone loss. 34

Furthermore, the nationality-wise differences in overall mean bone loss among diabetic and nondiabetic patients were higher in Saudi diabetic patients, but the differences were not significant. The non-Saudis nationals were mainly from Pakistan, India, Bangladesh, and Philippines. Delgado-Angulo et al discovered a complex link between ethnicity, socioeconomic status, and PD. 35 The role of variables in determining ethnic disparities in oral health, however, has not been assessed. 35 Further research should be conducted to examine the comparative roles of various factors that might aid in identifying variables which are more appealing to take necessary actions to reduce ethnic disparities in adult oral health care. 35

The gender-related differences in mean alveolar bone loss among diabetic and nondiabetic patients were found to be significant for male patients but nonsignificant for female patients in this study. These findings are consistent with previous research that found males to be more likely to suffer from alveolar bone loss or periodontitis. 14 40 The causes of these gender differences are unknown; however, they may be related to males' poor compliance with oral hygiene practices. 43 44

Our study's limitations were that our sample size was relatively small and the fact that we only studied patients who visited a single dental hospital. As a result, future multicenter studies should be conducted, with a larger study population. In addition, the relationship between clinical parameters of periodontitis such as periodontal probing depth (PPD) and CAL was not investigated in this study. More research is needed to determine the relationship between clinical parameters (PPD and CAL), DM severity, and radiographic alveolar crestal bone loss.

## Conclusion

In our study population, the overall mean alveolar bone loss prevalence was greater in diabetic patients than in nondiabetic individuals. According to the severity of bone loss, the distribution of moderate and severe alveolar bone loss was higher in diabetic as compared with nondiabetic patients. Oral complications of DM are numerous and alveolar bone loss or PD is one of them. It is possible to avoid PD and enhance patients' quality of life by raising awareness and education among patients regarding the relationship between DM and oral health. Diabetic patients should be motivated to practice good oral hygiene and manage their blood sugar levels appropriately to prevent oral problems.

## References

[1] Saeedi P Petersohn I Salpea P Global and regional diabetes prevalence estimates for 2019 and projections for 2030 and 2045: Results from the International Diabetes Federation Diabetes Atlas, 9th edition Diabetes Res Clin Pract 2019 157 107843 https://doi.org/10.1016/j.diabres.2019.107843 31518657 10.1016/j.diabres.2019.107843

[2] Ali Hassan S Pratyusha F Diabetes and oral diseases- a review IP J Nutr Metab Heal Sci 2020 3 01 6 9

[3] Al-Maskari A Y Al-Maskari M Y Al-Sudairy S Oral manifestations and complications of diabetes mellitus: a review Sultan Qaboos Univ Med J 2011 11 02 179 186 https://pubmed.ncbi.nlm.nih.gov/21969888/21969888 PMC3121021

[4] Ramachandran A Know the signs and symptoms of diabetes Indian J Med Res 2014 140 05 579 581 https://www.ncbi.nlm.nih.gov/pmc/articles/PMC4311308/25579136 PMC4311308

[5] Aljulifi M Z Prevalence and reasons of increased type 2 diabetes in Gulf Cooperation Council Countries Saudi Med J 2021 42 05 481 490 33896777 10.15537/smj.2021.42.5.20200676 PMC9149705

[6] Al-Quwaidhi A J Pearce M S Sobngwi E Critchley J A O'Flaherty M Comparison of type 2 diabetes prevalence estimates in Saudi Arabia from a validated Markov model against the International Diabetes Federation and other modelling studies Diabetes Res Clin Pract 2014 103 03 496 503 24447810 10.1016/j.diabres.2013.12.036 PMC4013554

[7] Stumvoll M Goldstein B J van Haeften T W Type 2 diabetes: principles of pathogenesis and therapy Lancet 2005 365(9467):1333 1346 15823385 10.1016/S0140-6736(05)61032-X

[8] Indurkar M S Maurya A S Indurkar S Oral manifestations of diabetes Clin Diabetes 2016 34 01 54 57 26807010 10.2337/diaclin.34.1.54 PMC4714722

[9] Orbak R Simsek S Orbak Z Kavrut F Colak M The influence of type-1 diabetes mellitus on dentition and oral health in children and adolescents Yonsei Med J 2008 49 03 357 365 18581583 10.3349/ymj.2008.49.3.357 PMC2615350

[10] Graves D T Li J Cochran D L Inflammation and uncoupling as mechanisms of periodontal bone loss J Dent Res 2011 90 02 143 153 21135192 10.1177/0022034510385236 PMC3144100

[11] Borrell L N Papapanou P N Analytical epidemiology of periodontitis J Clin Periodontol 2005 32 06 132 158 16128835 10.1111/j.1600-051X.2005.00799.x

[12] Genco R J Current view of risk factors for periodontal diseases J Periodontol 1996 67 10 1041 1049 10.1902/jop.1996.67.10.1041 8910821

[13] Genco R J Graziani F Hasturk H Effects of periodontal disease on glycemic control, complications, and incidence of diabetes mellitus Periodontol 2000 2020 83 01 59 65 32385875 10.1111/prd.12271

[14] Madi M Tabasum A Elakel A Periodontal risk assessment in a teaching hospital population in Saudi Arabia's Eastern Province Saudi Dent J 2021 33 08 853 859 34938025 10.1016/j.sdentj.2021.09.014 PMC8665182

[15] Nazir M A AlGhamdi L AlKadi M AlBeajan N AlRashoudi L AlHussan M The burden of diabetes, its oral complications and their prevention and management Open Access Maced J Med Sci 2018 6 08 1545 1553 30159091 10.3889/oamjms.2018.294 PMC6108795

[16] Gupta N Gupta N D Gupta A Goyal L Garg S The influence of type 2 diabetes mellitus on salivary matrix metalloproteinase-8 levels and periodontal parameters: a study in an Indian population Eur J Dent 2015 9 03 319 323 26430357 10.4103/1305-7456.163222 PMC4569980

[17] Graves D T Oates T Garlet G P Review of osteoimmunology and the host response in endodontic and periodontal lesions J Oral Microbiol 2011 3 3 10.3402/jom.v3i0.5304 PMC3087239 21547019

[18] working group 2 of the joint EFP/AAP workshop Chapple I L Genco R Diabetes and periodontal diseases: consensus report of the Joint EFP/AAP Workshop on Periodontitis and Systemic Diseases J Periodontol 2013 84(4, Suppl):S106 S112 23631572 10.1902/jop.2013.1340011

[19] Wu Y Y Xiao E Graves D T Diabetes mellitus related bone metabolism and periodontal disease Int J Oral Sci 2015 7 02 63 72 25857702 10.1038/ijos.2015.2 PMC4817554

[20] Preshaw P M Alba A L Herrera D Periodontitis and diabetes: a two-way relationship Diabetologia 2012 55 01 21 31 22057194 10.1007/s00125-011-2342-y PMC3228943

[21] Heji E S Bukhari A A Bahammam M A Homeida L A Aboalshamat K T Aldahlawi S A Periodontal disease as a predictor of undiagnosed diabetes or prediabetes in dental patients Eur J Dent 2021 15 02 216 221 33285572 10.1055/s-0040-1719208 PMC8184281

[22] Matrooshi K A Raeesi S A Tawfik A R Knowledge of physicians about the interrelationship between diabetes mellitus and periodontitis in the United Arab Emirates Eur J Dent 2022 10.1055/s-0042-1746413 PMC9949970 35817089

[23] Ivanauskaite D Lindh C Rangne K Rohlin M Comparison between Scanora panoramic radiography and bitewing radiography in the assessment of marginal bone tissue Stomatologija 2006 8 01 9 15 16687909

[24] Akesson L Rohlin M Håkansson J Håkansson H Näsström K Comparison between panoramic and posterior bitewing radiography in the diagnosis of periodontal bone loss J Dent 1989 17 06 266 271 2607022 10.1016/0300-5712(89)90031-6

[25] Llambés F Arias-Herrera S Caffesse R Relationship between diabetes and periodontal infection World J Diabetes 2015 6 07 927 935 26185600 10.4239/wjd.v6.i7.927 PMC4499526

[26] Artese H PC Foz A M Rabelo MdeS Periodontal therapy and systemic inflammation in type 2 diabetes mellitus: a meta-analysis PLoS One 2015 10 05 e0128344 10.1371/journal.pone.0128344 26010492 PMC4444100

[27] Maboudi A Akha O Heidari M Mohammadpour R A Gheblenama P Shiva A Relation between periodontitis and prediabetic condition J Dent (Shiraz) 2019 20 02 83 89 31214634 10.30476/DENTJODS.2019.44928 PMC6538893

[28] Seppälä B Seppälä M Ainamo J A longitudinal study on insulin-dependent diabetes mellitus and periodontal disease J Clin Periodontol 1993 20 03 161 165 8450080 10.1111/j.1600-051x.1993.tb00338.x

[29] Stegeman C A Oral manifestations of diabetes Home Healthc Nurse 2005 23 04 233 240, quiz 241–242 15824612 10.1097/00004045-200504000-00009

[30] Kaur G Mohindra K Singla S Autoimmunity-basics and link with periodontal disease Autoimmun Rev 2017 16 01 64 71 27664383 10.1016/j.autrev.2016.09.013

[31] Koutouzis T Haber D Shaddox L Aukhil I Wallet S M Autoreactivity of serum immunoglobulin to periodontal tissue components: a pilot study J Periodontol 2009 80 04 625 633 19335083 10.1902/jop.2009.080422 PMC3594842

[32] Kudiyirickal M G Pappachan J M Diabetes mellitus and oral health Endocrine 2015 49 01 27 34 25487035 10.1007/s12020-014-0496-3

[33] Li Q Hao S Fang J Xie J Kong X H Yang J X Effect of non-surgical periodontal treatment on glycemic control of patients with diabetes: a meta-analysis of randomized controlled trials Trials 2015 16 01 291 10.1186/s13063-015-0810-2 26137892 PMC4490675

[34] Hossain M Z Fageeh H N Elagib M F Prevalence of periodontal diseases among patients attending the Outpatient Department at the College of Dentistry, King Khalid University, Abha, Saudi Arabia City Dent Coll J 2013Feb;10 01 9 12

[35] Delgado-Angulo E K Bernabé E Marcenes W Ethnic inequalities in periodontal disease among British adults J Clin Periodontol 2016 43 11 926 933 27461047 10.1111/jcpe.12605

[36] Cruz G D Chen Y Salazar C R Le Geros R Z The association of immigration and acculturation attributes with oral health among immigrants in New York City Am J Public Health 2009 99 02 S474 S480 19443820 10.2105/AJPH.2008.149799 PMC4504384

[37] Sanders A E A Latino advantage in oral health-related quality of life is modified by nativity status Soc Sci Med 2010 71 01 205 211 20434250 10.1016/j.socscimed.2010.03.031 PMC2885514

[38] Borrell L N Taylor G W Borgnakke W S Factors influencing the effect of race on established periodontitis prevalence J Public Health Dent 2003 63 01 20 29 12597582 10.1111/j.1752-7325.2003.tb03470.x

[39] Craig R G Boylan R Yip J Prevalence and risk indicators for destructive periodontal diseases in 3 urban American minority populations J Clin Periodontol 2001 28 06 524 535 11350519 10.1034/j.1600-051x.2001.028006524.x

[40] Gilbert G H Racial and socioeconomic disparities in health from population-based research to practice-based research: the example of oral health J Dent Educ 2005 69 09 1003 1014 16141086

[41] Borrell L N Crawford N D Social disparities in periodontitis among US adults: the effect of allostatic load J Epidemiol Community Health 2011 65 02 144 149 19996354 10.1136/jech.2009.098269

[42] Borrell L N Taylor G W Borgnakke W S Woolfolk M W Nyquist L V Perception of general and oral health in White and African American adults: assessing the effect of neighborhood socioeconomic conditions Community Dent Oral Epidemiol 2004 32 05 363 373 15341621 10.1111/j.1600-0528.2004.00177.x

[43] Albandar J M Kingman A Gingival recession, gingival bleeding, and dental calculus in adults 30 years of age and older in the United States, 1988-1994 J Periodontol 1999 70 01 30 43 10052768 10.1902/jop.1999.70.1.30

[44] Slade G D Spencer A J Periodontal attachment loss among adults aged 60+ in South Australia Community Dent Oral Epidemiol 1995 23 04 237 242 7587146 10.1111/j.1600-0528.1995.tb00238.x

